# Synergetic engineering of *Escherichia coli* for efficient production of l-tyrosine

**DOI:** 10.1016/j.synbio.2023.10.005

**Published:** 2023-11-07

**Authors:** Jurong Ping, Lian Wang, Zhijie Qin, Zhemin Zhou, Jingwen Zhou

**Affiliations:** a Engineering Research Center of Ministry of Education on Food Synthetic Biotechnology, Jiangnan University, 1800 Lihu Road, Wuxi, Jiangsu, 214122, China; b Science Center for Future Foods, Jiangnan University, 1800 Lihu Road, Wuxi, Jiangsu, 214122, China; c Key Laboratory of Industrial Biotechnology, Ministry of Education and School of Biotechnology, Jiangnan University, 1800 Lihu Road, Wuxi, Jiangsu, 214122, China; d Jiangsu Province Engineering Research Center of Food Synthetic Biotechnology, Jiangnan University, Wuxi, 214122, China

**Keywords:** L-tyrosine, Cofactor regeneration, Phosphoketolase pathway, *Escherichia coli*

## Abstract

l-Tyrosine, an aromatic non-essential amino acid, is the raw material for many important chemical products, including levodopa, resveratrol, and hydroxytyrosol. It is widely used in the food, drug, and chemical industries. There are many studies on the synthesis of l-tyrosine by microorganisms, however, the low titer of l-tyrosine limited the industrial large-scale production. In order to enhance l-tyrosine production in *Escherichia coli*, the expression of key enzymes in the shikimate pathway was up- or down-regulated. The l-tyrosine transport system and the acetic acid biosynthesis pathway were modified to further enhance l-tyrosine production. In addition, the phosphoketolase pathway was introduced in combination with cofactor engineering to redirect carbon flux to the shikimate pathway. Finally, after adaptive laboratory evolution to low pH an optimal strain was obtained. The strain can produce 92.5 g/L of l-tyrosine in a 5-L fermenter in 62 h, with a yield of 0.266 g/g glucose.

## Graphical abstract

**Figure:**
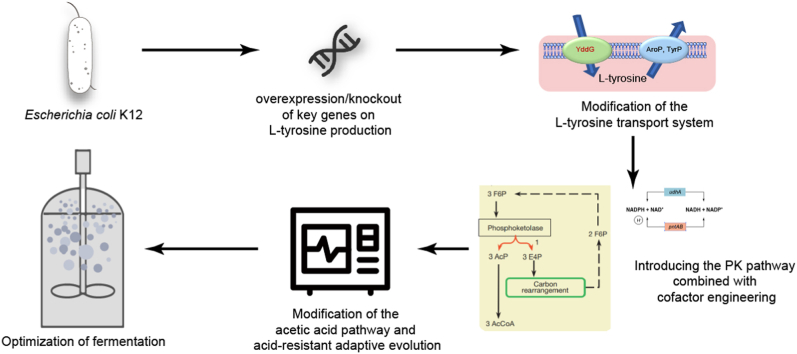

## 1 Introduction

l-Tyrosine is one of the 20 amino acids used to synthesize proteins, which is widely used in the food, feed, chemical and pharmaceutical industries. It is the precursor of a variety of natural products, including caffeic acid, flavonoids, curcumin [1,2], 3,4-dihydroxy-l-phenylalanine (l-DOPA), *p*-hydroxystyrene and *p*-hydroxycinnamic acid [[3], [4], [5]]. The market demand for l-tyrosine continues to rise [6,7], with a high market price about 80,000 CNY per ton. The traditional production methods of l-tyrosine include protein hydrolysis, chemical synthesis, enzymatic methods, and microbial fermentation [8,9]. Protein hydrolysis and chemical synthesis methods have been phased out, and the main production methods at present are enzymatic and microbial fermentation methods.

The enzymatic method is based on a reaction in which β-tyrosinase converts substrates such as phenol-l-serine or phenol-pyruvate-ammonia into l-tyrosine. The disadvantages are high costs and poor stability. Furthermore, the phenol used in the production process is highly toxic, corrosive, and extremely harmful to the environment [10]. Microbial fermentation uses simple carbon sources as raw materials, and its advantages include low cost, a simple process, and a low environmental burden [11]. The *de novo* synthesis of l-tyrosine by microorganisms is considered to have very broad development prospects. Despite extensive research on the shikimate pathway over the past 40 years [12,13], the use of microorganisms to synthesize l-tyrosine still faces many challenges, such as the low metabolic flux of the shikimate pathway, and the manipulation of genes involves complex metabolic regulation. During fed-batch fermentation processes, the strains are subjected to high-density and high-intensity production for a prolonged period. The lack of persistence and stability of strains is an important limiting factor for industrialization. Currently, with the development of metabolic engineering and biotechnology, it has gradually become easier and more feasible to rationally design microbial metabolic pathways to achieve high accumulation of l-tyrosine [14,15].

Strategies for enhancing l-tyrosine accumulation have mainly focused on increasing the flux of the shikimate pathway, including increasing the precursor supply, increase the expression level of intermediate enzymes, and releasing feedback inhibition [16]. The most effective strategy for accumulating l-tyrosine is to express *aroG*^*fbr*^(D146 N) and *tyrA*^*fbr*^(M53I/A354V). AroG can catalyze the shikimate pathway precursor PEP (Phosphoenolpyruvate) and E4P (d-erythrose 4-phosphate) to produce DAHP [17], while TyrA is a critical enzyme that catelyzes the production of l-tyrosine by chorismite [18]. On the basis of the above strategies, by relieving the feedback repression effect of *tyrR* on shikimate pathway enzymes [19], an engineered strain (HRP) was previously obtained, which can accumulate 55.54 g/L of l-tyrosine [20]. Other methods were also used, including increasing the supply of precursors by increasing *ppsA*(phosphoenolpyruvate synthetase) and *tktA*(transketolase 1) expression [18], knocking out *pheA*(fused chorismate mutase/prephenate dehydratase) and other genes to block branch metabolism [21], and identifying the key enzymes of the shikimate pathway through combined expression verification [22].

Though many methods have been attempted, the production of l-tyrosine is still limited. In the present study, the reported l-tyrosine production methods were evaluated comprehensively, and then combination validation was carried out. To further improve the accumulation of l-tyrosine, the l-tyrosine transport system was modified to reduce the intracellular accumulation of l-tyrosine. Then the phosphoketolase (PK) pathway was introduced and cofactor engineering was performed to direct glucose metabolism to the shikimate pathway for l-tyrosine synthesis. Additionally, to enhance l-tyrosine accumulation in the middle and late stages of fermentation, the acetic acid pathway of *Escherichia coli* was modified, and tolerance of the obtained strain to acetic acid was improved by adaptive evolution. Finally, through systematic fermentation optimization in a 5-L fermenter, 92.5 g/L of l-tyrosine was obtained with the engineered strain (HGD), which is the highest titer reported so far.

## 2 Materials and methods

### 2.1 Strains, media, and chemicals

The strains used in this study are listed in Table 1. The l-tyrosine-deficient strain WSH-Z06 (MG1655) stored in our laboratory was selected to produce l-tyrosine [23]. The plasmid was constructed using *E. coli* JM109 and *E. coli* BL21(DE3) as clone hosts. The recombinant *E. coli* genome was modified by the CRISPR/Cas9 system [24]. *E. coli* strains were cultured with Luria–Bertani (LB) for DNA manipulation and protein screening. Kanamycin or streptomycin was added as required.

**Table 1:** Strains used in this study.

Strains	Description	Sources
*E. coli* JM109	Cloning strain	Takara Bio
*E. coli* WSH-Z06	l-Tyriosine auxotrophic	[23]
HGA1	*E. coli* WSH-Z06 Δ*tyrR, pheA and trpE,* containing pAP11	This study
HGA2	*E. coli* WSH-Z06 Δ*tyrR, pheA and adhE* containing pAP11	This study
HGA3	*E. coli* WSH-Z06 Δ*tyrR, pheA and ldhA* containing pAP11	This study
HGA4	*E. coli* WSH-Z06 Δ*tyrR, pheA and trpE* containing pAP12	This study
HGA5	*E. coli* WSH-Z06 Δ*tyrR, pheA and adhE,* containing pAP12	This study
HGA6	*E. coli* WSH-Z06 Δ*tyrR, pheA and ldhA,* containing pAP12	This study
HGB1	HGA1 Δ*aroP*, containing pAP11	This study
HGB2	HGA1 Δ*tyrP*, containing pAP11	This study
HGB3	HGA1 Δ*aroP* and *tyrP*, containing pAP11	This study
HGB4	HGA1 Δ*aroP*, containing pAP31	This study
HGB5	HGA1 Δ*tyrP*, containing pAP31	This study
HGB6	HGA1 Δ*aroP* and *tyrP*, containing pAP31	This study
HGC1	HGB6 *dadX-cvrA*::P_j231119_-*yddG*, *tyrP*::P_j231119_-*tktA*, *trpE*::P_j231119_-*ppsA*, containing pAP11	This study
HGC2	HGC1 containing pAP32	This study
HGC3	HGC1 containing pAP33	This study
HGC4	HGC3 *ykgH-betA*::P_j231119_-*udhA*, *yeeJ-yeeL*::P_j231119_-*pntAB*, containing pAP33	This study
HGD1	HGC4 Δ*ackA*, containing pAP33	This study
HGD2	HGC4 Δ*pta*, containing pAP32	This study
HGD3	HGC4 Δ*poxB*, containing pAP33	This study
HGD (M9)	HGD3 with evolution of acid resistance	This study

l-Tyrosine production medium [20] contained glucose 35 g L^−1^, (NH_4_)_2_SO_4_ 5 g L^−1^, tryptone 4 g L^−1^, KH_2_PO_4_ 3 g L^−1^, MgSO_4_·7H_2_O 3 g L^−1^, NaCl 1 g L^−1^, Na_3_C_6_H_5_O_7_·2H_2_O 1.5 g L^−1^, CaCl_2_·2H_2_O 0.015 g L^−1^, FeSO_4_·7H_2_O 0.1125 g L^−1^, vitamin B1 0.075 g L^−1^, yeast extract 2 g L^−1^, and 0.3 % (v/v) trace element solution (TES). When preparing the medium, glucose, MgSO_4_·7H_2_O, CaCl_2_·2H_2_O, FeSO_4_·7H_2_O, and vitamin B1 were sterilized through membrane filtration (0.22 μm). The remaining ingredients were prepared in the fermentation tank and sterilized at 115 °C for 20 min. TES contained Al_2_(SO_4_)_3_·18H_2_O 2.0 g L^−1^, CoSO_4_·7H_2_O 0.75 g L^−1^, CuSO_4_·5H_2_O 2.5 g L^−1^, H_3_BO_3_ 0.5 g L^−1^, MnSO_4_·H_2_O 24 g L^−1^, NiSO_4_·6H_2_O 2.5 g L^−1^, and ZnSO_4_·7H_2_O 15 g L^−1^.

### 2.2 Plasmid construction and genome modification

The heat-inducible plasmid framework was obtained from the pAP-B03 plasmid [23], including the kanamycin gene, the P_R_P_L_ promoter, and the p15A replicon. The *aroG*^*fbr*^ and *tyrA*^*fbr*^ genes were amplified from the pCDF plasmid [25]. As such, pAP-*aroG*^*fbr*^-*tyrA*^*fbr*^ was assembled. The upregulated genes *yddG*, *aroA*, *aroB*, *aroC*, *aroE*, *aroL*, *tktA*, and *ppsA* were amplified from the genomic DNA of *E. coli* K12, and the genes was ligated with the plasmid pAP-*aroG*^*fbr*^-*tyrA*^*fbr*^. The plasmid and primer sequences are listed in Table 2 and S2.

**Table 2:** Plasmids used in this study.

Plasmids	Description	Sources
pAP-B03	Carring *pheA*^*fbr*^ and *aroF*^*wt*^ genes, p15A ori with P_R_P_L_;kan^R^	Our laboratory
pCDF-*aroG*^*fbr*^-*tyrA*^*fbr*^	pCDFDuet-1:*aroG*^*fbr*^::*tyrA*^*fbr*^, Str^R^	Our laboratory
*pta*rget	*pMB1*,*aadA*,sgRNA-Blank,Spc^R^	[26]
pCas9	*repA*101(Ts) *kan* P*cas*-*cas9* P*araB*-*Red lacI* P*trc*-sgRNA-*pMB1*	[26]
pAP01	pAP with *aroG*^*fbr*^ and *tyrA*^*fbr*^, p15A ori with P_R_P_L_;kan^R^	This study
pAP11	pAP with *aroG*^*fbr*^, *tyrA*^*fbr*^, *tkt* *A* and *ppsA*, p15A ori with P_R_P_L_;kan^R^	This study
pAP12	pAP with *aroG*^*fbr*^, *tyrA*^*fbr*^ and *aroABC*, p15A ori with P_R_P_L_;kan^R^	This study
pAP13	pAP with *aroG*^*fbr*^, *tyrA*^*fbr*^ and *aroBEL*, p15A ori with P_R_P_L_;kan^R^	This study
pAP21	pAP with *aroG*^*fbr*^, *tyrA*^*fbr*^, *tktA*, *ppsA* and *aroABC*, p15A ori with P_R_P_L_;kan^R^	This study
pAP22	pAP with *aroG*^*fbr*^, *tyrA*^*fbr*^, *tktA*, *ppsA* and *aroBEL*, p15A ori with P_R_P_L_;kan^R^	This study
pAP31	pAP with *aroG*^*fbr*^, *tyrA*^*fbr*^, *tktA*, *ppsA* and *yddG*, p15A ori with P_R_P_L_;kan^R^	This study
pAP32	pAP with *aroG*^*fbr*^, *tyrA*^*fbr*^ and *fpk*, p15A ori with P_R_P_L_;kan^R^	This study
pAP33	pAP with *aroG*^*fbr*^, *tyrA*^*fbr*^, *fpk* and *pta*, p15A ori with P_R_P_L_;kan^R^	This study

CRISPR/Cas9 gene editing technology [26] was used to knock out, integrate, and replace target genes. In order to knock out *tyrR*, *pheA*, *trpE*, *aroP*, *tyrP*, and *poxB*, the pTarget plasmid with N20 sequence (https://benchling.com/editor) and homologous arm knockout box was transformed into competent cells by electroporation. Cells were cultured overnight at 30 °C and the successful knockout of genes was verified through colony PCR and sequencing. The pTarget plasmid and pCas plasmid were discarded by adding 10 mM rhamnose and 5 g/L sucrose respectively.

### 2.3 Culture conditions

The stored *E. coli* was activated on a LB plate for 12 h. After overnight culture, the cells were transferred to a 10-mL tube containing 2 mL LB culture medium, and the primary seed solution was obtained by incubating at 33 °C and 220 rpm for 10 h. Then the solution was inoculated to a 250-mL shake flask containing 50 mL LB culture medium and incubated at 33 °C and 220 rpm for 10 h. Then the secondary seed solution was transferred to a 5-L fermenter containing 2 L of fermentation medium. The fermentation temperature was 33 °C. When the cell concentration reached OD_600_ = 20–23, the temperature was raised to 38 °C to induce the production of l-tyrosine, and 750 g/L glucose was added to control the glucose concentration in the medium.

### 2.4 Analytical methods

The acetic acid content was detected by HPLC. The fermentation broth was centrifuged at 12,000×*g* for 3 min, and the supernatant was diluted 10 times before passing through the membrane. An Agilent 1260 high-performance liquid chromatograph was used for detection. A Bio-Rad Aminex HPX-87H chromatographic column and a parallax refractive detector were used. The mobile phase was 0.005 M H_2_SO_4_ and the flow rate was 0.5 mL/min.

One milliliter of fermentation broth was taken out, and 6 M HCl (hydrochloric acid) was used to dilute the sample and remove residual CaCO_3_. After thorough mixing, OD_600_ was measured with HCl as the control. The sample was centrifuged at 12,000×*g* for 3 min. The supernatant was collected, and the filtered fermentation broth was used for HPLC determination.

The content of l-tyrosine was determined by HPLC. The injection volume was 10 μL, the mobile phase was 0.1 M sodium acetate and methanol (90:10, v/v). The detection wavelength of the UV detector was 280 nm, the flow rate was 1.0 mL/min, the column temperature was 30 °C.

### 2.5 Adaptive evolution to low pH conditions

The acid-resistant adaptive evolution of the strain was completed with a Microbial Microdroplet Culture (MMC) system [27]. *E. coli* HGD3 was taken from glycerol tubes and activated on kanamycin plates at 37 °C for 12 h. A single colony was inoculated into a pH 6.9 LB medium and incubated at 37 °C and 220 rpm for 12 h. The seed solution was transferred to a specialized sterile bottle. A pH 6.8 culture medium was transferred to a dedicated sterile culture medium bottle, and an adaptive evolution pH 5.1 culture medium was transferred to another dedicated sterile culture medium bottle. MMC adaptive evolution first forms a large number of droplets. The second step is the cultivation of microdroplets. After a period of adaptive evolution, droplets with high OD_600_ values were collected and dispersed on pH 5.1 plates under selective pressure.

## 3 Results and discussion

### 3.1 Effects of overexpression/knockout of key genes on l-tyrosine production

**Fig. 1:**
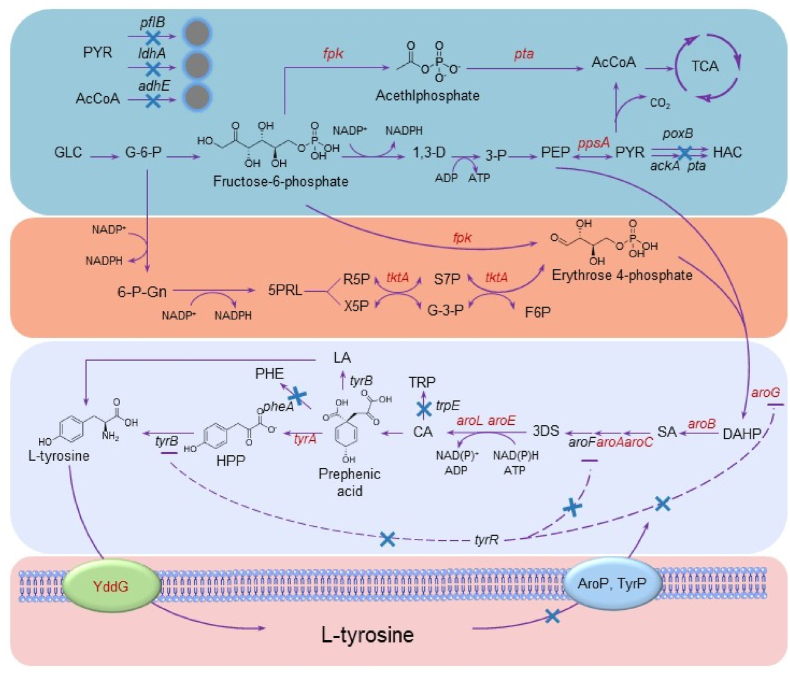
**Biosynthesis of****l****-tyrosine from glucose.** The first part of the diagram (blue area) is the glycolytic pathway. The second part of the diagram (orange area) is the pentose phosphate pathway. The third part of the figure (lavender part) is the synthesis of l-tyrosine by the shikimate pathway. The fourth part (bottom part) in the figure is a schematic diagram of the modification of the l-tyrosine transport system. The blue cross in the figure indicates the knockout or inactivation of the corresponding gene. Multiple arrows in the figure indicate multi-step reactions. Genes in red in the figure indicate overexpression. The dotted line in the figure represents repressive feedback inhibition. *aroG*, 3-deoxy-7-phosphoheptulonate synthase; *aroA*, 3-phosphoshikimate 1-carboxyvinyltransferase; *aroB*, 3-dehydroquinate synthase; *aroC*, 3-dehydroquinate synthase; *pta*, phosphate acetyltransferase; *fpk*, phosphoketolase; *poxB*, pyruvate oxidase; *tktA*, transketolase 1; *ppsA*, phosphoenolpyruvate synthetase; *tyrA*, fused chorismate mutase/prephenate dehydrogenase; *yddG*, amino acid exporter; *aroP*, aromatic amino acid:H(+) symporter; *tyrP*, tyrosine:H(+) symporter; *pheA*, fused chorismate mutase/prephenate dehydratase; *adhE*, fused acetaldehyde-CoA dehydrogenase and iron-dependent alcohol dehydrogenasealdehyde/alcohol dehydrogenase; *pflB*, pyruvate formate-lyase; *aroL*, shikimate kinase 2; *aroE*, shikimate dehydrogenase; *ldhA*, d-lactate dehydrogenase; *trpE*, anthranilate synthase subunit.

**Fig. 2:**
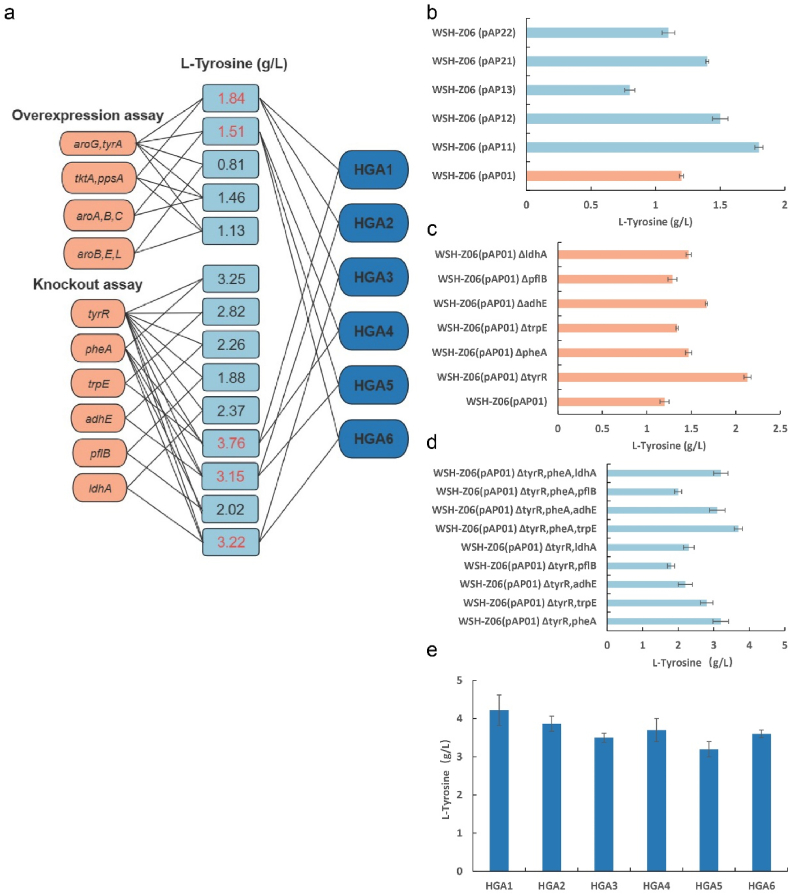
**Construction of****l****-tyrosine high-yielding strain.** (a) Schematic diagram of the combined pathways for the expression and knockout of l-tyrosine synthesis-related genes. (b) The results of 48-h shake flask fermentation of the original strain WSH-Z06 expressing l-tyrosine synthesis-related genes. (c) The original strain WSH-Z06 carries mutant *aroG* and *tyrA*. The 48-h shake flask fermentation results after knocking out relevant target genes individually are shown. (d) The results of 48-h shake flask fermentation of the original strain WSH-Z06 with combined knockout of related genes. (e) Combining the best results of the combined expression and knockout strategies, six strains were selected for 48-h shake flask fermentation.

*E. coli* WSH-Z06 was selected as the starting strain. Several genes related to the accumulation of shikimate and aromatic amino acids were screened, such as *aroG*, *tyrA*, *tktA*, *ppsA*, *aroA*, *aroB*, *aroC*, *aroE*, *aroL*, *tyrR*, *pheA*, *trpE*, *adhE*, *pflB*, and *ldhA* (Fig. 1). Knockout and/or overexpression of these genes may be beneficial for l-tyrosine accumulation (Fig. 2a). In the overexpression assay, the best results were obtained after combined expression of *aroG*^*fbr*^, *ppsA*, *tktA*, and *tyrA*^*fbr*^; the optimal strain could accumulate 1.84 g/L of l-tyrosine in a shake flask after 48 h (Fig. 2c). For the knockout assay, strain WSH-Z06 carrying mutant *aroG*^*fbr*^ and *tyrA*^*fbr*^ genes was used as the starting strain. After three rounds of knockout experiments, the combined knockout of *tyrR*, *pheA*, and *trpE* resulted in the highest l-tyrosine accumulation (3.76 g/L) in a shake flask after 48 h (Fig. 2d).

The phosphotransferase system (PTS) utilizes PEP to transport glucose. Modifying the PTS can augment the supply of PEP, therefore enhancing carbon flux through the shikimate pathway. This modification is also the preferred strategy to obtain a high l-tyrosine producer. However, alteration of PTS significantly inhibited the growth of the applied strain in this study (data not shown). From the results of the combination assay (Fig. 2a), it can be seen that the expression of *aroG*^*fbr*^ and *tyrA*^*fbr*^ increases the synthesis of l-tyrosine [17,28] and knockout of *tyrR* relieves the repression of some shikimate pathway enzymes [29]. These are the most used strategies for enhancing l-tyrosine production in *E. coli*. On this basis, the genes *ppsA* and *tktA* were expressed to increase the supply of phosphoenolpyruvate and erythrose 4-phosphate precursors of the shikimate pathway [30], the deletion of *trpE* blocked the branching metabolism from chorismic acid to tryptophan [31], and the deletion of *pheA* blocked the branching metabolism from prephenate to phenylalanine [32]. The final strain (HGA1) could produce 4.22 g/L of l-tyrosine in a shake flask after 48 h (Fig. 2e).

### 3.2 Modification of the l-tyrosine transport system

**Fig. 3:**
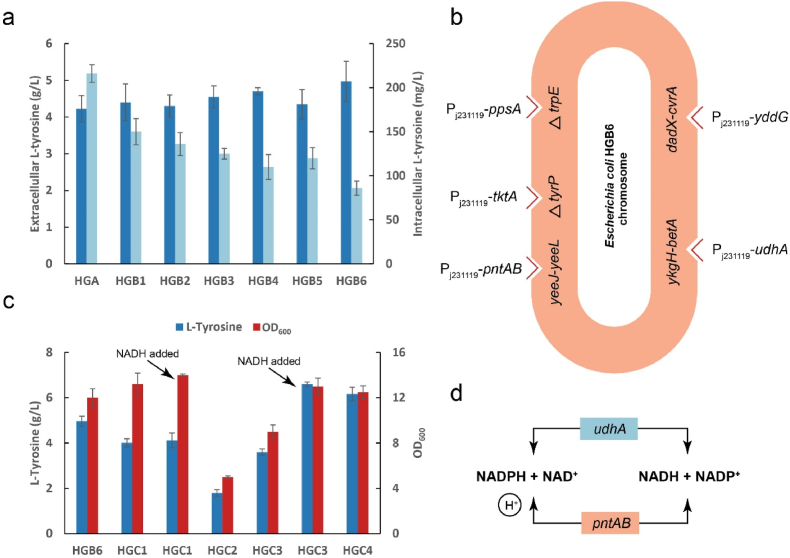
**Modification of the transport system and introduction of the PK pathway.** (a) Intra- and extracellular l-tyrosine levels of *E. coli* HGA with modified l-tyrosine transport system after 48-h shake flask fermentation. (b) Schematic diagram of the integration of some genes into the HGB6 genome to simplify the plasmid. (c) Growth and l-tyrosine accumulation of bacteria with exogenous introduction of the PK pathway combined with cofactor engineering, after 48-h fermentation in shake flasks. (d) Schematic diagram showing that the expression of endogenous *udhA* and *pntAB* enhances the supply of NAD(P)H in *E. coli*.

The l-tyrosine concentration in strain HGA1 cells was 942 % higher than in the control group. The intracellular overaccumulation of l-tyrosine may reduce cellular activity and affect the fermentation performance. Therefore, it is necessary to modify the l-tyrosine transport system of *E. coli*. The aromatic amino acid transporter AroP and the l-tyrosine-specific transporter TyrP can transport extracellular l-tyrosine into cells [33]. The aromatic amino acid exporter YddG can transfer aromatic amino acids from within the cell to the extracellular environment [34]. The simultaneous knockout of *aroP* and *tyrP* resulted in a 42.1 % decrease in intracellular l-tyrosine content. On this basis, the engineered strain HGB6 was obtained by expressing YddG, and the intracellular l-tyrosine content was further decreased by 60 % (Fig. 3a). At the same time, the extracellular accumulation of l-tyrosine was increased to 4.97 g/L, which was 17.8 % higher than that of HGA1.

Even though molecular biology and metabolic engineering technologies have become very mature, there are still many unexplained issues related to the complex metabolic regulation within bacteria [35]. In previous study, it was observed that cellular growth was inhibited when *tyrP* and *aroP* were knocked out consecutively [20]. In the present study, after knocking out *aroP*, *tyrP* was not immediately knocked out, and some other modifications were made. It was found that the strain could grow normally. Both *aroP* and *tyrP* encode putative membrane proteins involved in l-tyrosine transport. The consecutive knockout of two membrane proteins in a short period of time affects the stability of the cell membrane of *E. coli*, thereby affecting its growth [36]. After knocking out *aroP*, *E. coli* adapts to this transformation strategy through internal self-adjustment after a certain amount of time [37]. At this point, further knockout of *tyrP* will not have a significant impact on *E. coli* growth.

### 3.3 Introducing the PK pathway combined with cofactor engineering

To further improve the l-tyrosine accumulation of HGB6, the PK pathway was introduced to directionally guide glucose to the shikimate pathway [38]. In order to reduce the burden of plasmid on the strain, the endogenous genes *ppsA*, *tktA*, and *yddG* were linked to the strong constitutive promoter P_j231119_ and integrated into the HGB6 genome (Fig. 3b) [39]. The OD_600_ of strain HGC1 in a shake flask was increased by 9.8 %. Expressing phosphoketolase (*fpk*) from *Bifidobacterium adolescens* and endogenous phosphoacetyltransferase (*pta*) from *E. coli*, the accumulation of l-tyrosine significantly decreased. After the addition of cofactor NADH, the growth of the strain was restored and l-tyrosine accumulation increased to 6.6 g/L (Fig. 3c). Integrating the endogenous *udhA* and *pntAB* genes of *E. coli* with the promoter P_j231119_ into the genome, the mutual transformation between intracellular NADPH and NADH was increased (Fig. 3d) [40], resulting in strain HGC4, which could accumulate 6.17 g/L of l-tyrosine after 48 h.

In the HGC3 strain, both FPK and PTA are expressed simultaneously, leading to a slight restoration in growth, while there still remains a great gap compared with the HGC1 strain. In addition, the accumulation of l-tyrosine is also lower than that of HGC1 strain. Modifications of the glycolysis and shikimate pathways enhanced the flux through the shikimate pathway. However, following the introduction of FPK, some of the carbon metabolic flow is diverted directly from F6P to the shikimate pathway via the PK metabolic pathway, thereby further reducing the flux through the glycolysis pathway. Given that the glycolysis pathway is the primary source of NADH production [41], this alteration, coupled with the previous modifications, leads to an imbalance of cofactors in *E. coli*, which may account for the decline in l-tyrosine production. External addition of NADH during induction was found to significantly increase l-tyrosine accumulation.

### 3.4 Modification of the acetic acid pathway to shorten fermentation time

**Fig. 4:**
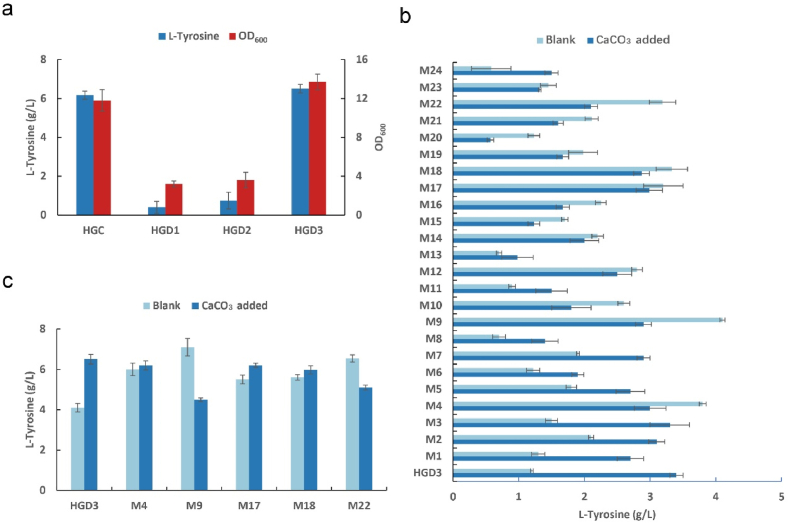
**Acetic acid system modification and acid resistance evolution of *E. coli*.** (a) Three genes related to acetic acid accumulation in *E. coli* (*ackA*, *pta*, and *poxB*) were knocked out. The growth and fermentation results after 48-h fermentation in shake flasks are shown. (b) After acid resistance evolution, 24 strains were selected for orifice plate fermentation verification and fermented for 48 h on fermentation medium with and without CaCO_3_ to verify the l-tyrosine accumulation ability. (c) Five l-tyrosine high-yielding strains, M4, M9, M17, M18, and M22, were screened from the orifice plate for shake flask fermentation verification and fermented on fermentation media with and without CaCO_3_ for 48 h to verify l-tyrosine accumulation.

HGC4 was fed-batch fermented in a 5-L fermenter, and 50.2 g/L of l-tyrosine could be accumulated in 69 h. However, 12.4 g/L acetic acid was detected in the fermentation broth (see Supplementary Materials). There are two main pathways for the production of acetate in *E. coli*, namely, the AckA–PTA pathway and the pyruvate oxidation pathway [42]. Knockout of the genes *ackA* and *pta* severely affected the growth of *E. coli*, and knockout of *poxB* resulted in a 33.6 % reduction in acetate levels and bacterial growth was slightly increased (Fig. 4a). In order to further improve the tolerance of HGD3 to acetic acid and enhance the activity of the strain in the later stage of fermentation, acid-resistant adaptive evolution of HGD3 was carried out. More than 200 droplets were screened for 50 passages, and finally 24 strains that grew well at pH 5.1 were screened out. The production of l-tyrosine by the screened strains was verified on orifice plates and in shaking flasks (Fig. 4b).

The accumulation of acetic acid is a major problem during the fermentation process of recombinant *E. coli*, which can inhibit cell growth and affect the accumulation of l-tyrosine [43,44]. AckA and PTA catalyze the conversion of acetate to acetyl-CoA; this reaction is reversible and is the main pathway for *E. coli* to produce acetate [45]. However, this pathway could not be modified due to its serious impact on bacterial growth. To verify the acid-resistant evolved strains, the acid-resistant bacteria were fermented in shake flasks with and without CaCO_3_ as a pH buffer and screened. Most of the screened bacteria could grow normally in the environment without CaCO_3_ as pH buffer. By rescreening the high-yielding strains screened from the orifice plates in a shaking flask, it could be seen that the increase in OD_600_ has a significant effect on the accumulation of l-tyrosine, and 7.11 g/L of l-tyrosine could be accumulated in the shake flask at 48 h (Fig. 4c).

### 3.5 Optimization of fermentation strategies in a 5-L fermenter

**Fig. 5:**
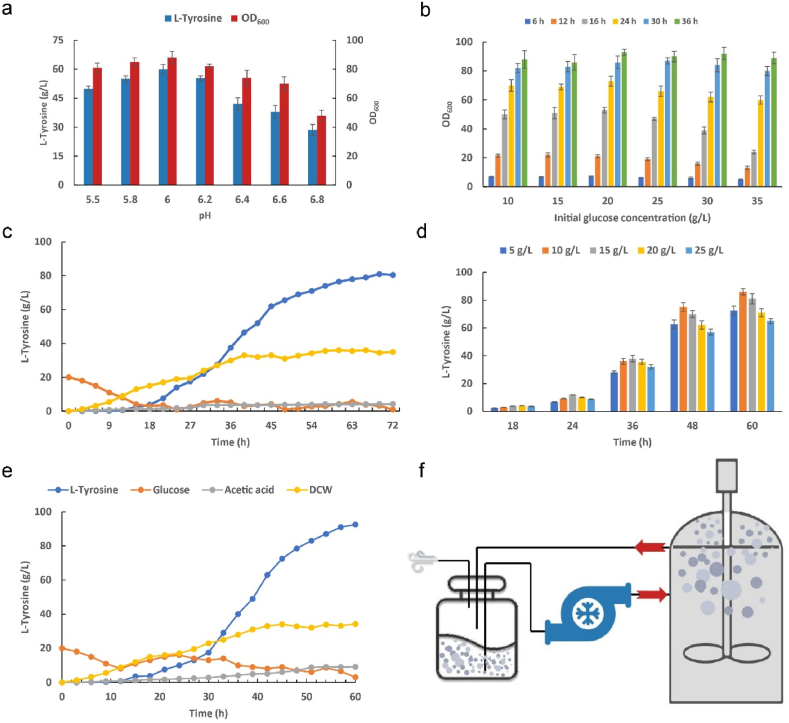
**Fermentation optimization in a 5-L fermenter.** (a) The pH of *E. coli* HGD obtained by acid resistance evolution was optimized. By analyzing the growth and l-tyrosine accumulation of the strain after 72 h, the optimal fermentation pH was determined to be 6.0. (b) In order to improve the growth of the strain before induction, the initial glucose concentration in the fermentation medium was optimized, and the growth of the strain was detected every 6 h from 6 h to 36 h. (c) In order to increase the readability of charts, DCW (g/L) is used here to represent the growth of the bacteria, DCW = OD_600_ × 0.364. The growth, acetic acid accumulation, and l-tyrosine accumulation of strain HGD after 72 h fermentation under optimal pH, initial glucose concentration, and dissolved oxygen content. (d) The glucose feeding rate during the fermentation process was adjusted, and the effect of different glucose concentrations on the accumulation of l-tyrosine at different stages of fermentation was tested. (e) Results after 62 h fermentation of HGD after optimizing pH, induction temperature, dissolved oxygen content, and glucose feed rate. (f) Schematic diagram of the reflux device.

The fermentation performance of HGD was tested. First, the temperature and pH of the fermentation were optimized. As shown in Fig. 5a, the pH value was 6.0 and the culture was kept at 33 °C until OD_600_ ≥ 20; then the temperature was changed to 38 °C to induce the production of l-tyrosine. During the fermentation process, it was found that it often takes 16 h or more from inoculation to reach OD_600_ = 20, which greatly prolongs the fermentation time. We tried to reduce the initial glucose concentration and increase the dissolved oxygen content to improve the early growth of *E. coli*. It was found that when the initial concentration of glucose was adjusted to 20 g/L and the dissolved oxygen content was controlled at 30 %, the OD_600_ could reach 20 in about 12 h (Fig. 5b). The glucose concentration in the fermenter was kept at 2–5 g/L during the fermentation process. Finally, 80.5 g/L of l-tyrosine could be accumulated in 72 h (Fig. 5c), with a yield of 0.278 g/g glucose.

When analyzing the fermentation broth, it was found that the concentration of acetic acid in the fermenter was not more than 4 g/L. Since HGD has undergone acid resistance evolution, we hypothesized it was possible to increase the glucose feed rate to shorten the fermentation time. In order to further explore the fermentation performance of HGD, we gradually increased the concentration of glucose. Finally, we controlled the glucose concentration at 10–15 g/L in the first 30 h of fermentation and at 5–10 g/L thereafter (Fig. 5d) to maximize the fermentation performance of HGD, which could accumulate 92.8 g/L of l-tyrosine in 62 h (Fig. 5e), with a yield of 0.266 g/g glucose. After fermentation, the acetic acid level was 9.0 g/L. Although this is relatively high, it accumulated almost linearly, so it did not affect the growth of *E. coli* in the pre- and mid-fermentation period.

Due to the fact that l-tyrosine is almost insoluble in neutral water [46], serious tank spraying problems occurred during the fermentation process. Broth is collected in sterile bottles and pumped back to the fermenter (Fig. 5f). At the end of this study, the feeding rate of the fermentation process was regulated, and a maximum l-tyrosine content of 92.8 g/L was achieved. Although the glucose conversion rate was decreased by 4.3 %, the l-tyrosine yield was improved by 12.3 g/L and the fermentation time was reduced by 10 h, which is of great significance for the industrial production of l-tyrosine [47].

## 4 Conclusions

In this study, the l-tyrosine synthesis pathway in *E. coli* was first comprehensively modified, followed by modifications to the acetic acid system and the l-tyrosine transport system. The PK pathway was introduced and combined with cofactor engineering to further enhance l-tyrosine accumulation. The engineered strains were subjected to acid-resistant adaptive evolution to improve the tolerance to acetic acid. Finally, through fermentation optimization, 92.5 g/L of l-tyrosine could be accumulated within 62 h. This study has great significance for the industrial production of l-tyrosine.

## CRediT authorship contribution statement

**Jurong Ping:** Investigation, Visualization, Writing – original draft, Writing – review & editing. **Lian Wang:** Investigation. **Zhijie Qin:** Writing – review & editing. **Zhemin Zhou:** Writing – review & editing. **Jingwen Zhou:** Supervision, Funding acquisition, Writing – review & editing.

## Declaration of competing interest

The authors declare that they have no known competing financial interests or personal relationships that could have appeared to influence the work reported in this paper.
