# Supplementary material for: Synergetic engineering of Escherichia coli for efficient production of l-tyrosine
Source: Synth Syst Biotechnol. 2023 Nov 7;8(4):724–31. doi: 10.1016/j.synbio.2023.10.005 (PMC10686809; doi:10.1016/j.synbio.2023.10.005)
Supplement: Multimedia component 1 [file mmc1.docx]

**Supplementary Information**

# Synergetic engineering of *Escherichia coli* for efficient production of L-tyrosine

Jurong Ping^1,2^, Lian Wang^1,2,3^, Zhijie Qin^1,2^, Zhemin Zhou^3,*^, Jingwen Zhou^1,2,3,4,*^

^1^ Engineering Research Center of Ministry of Education on Food Synthetic Biotechnology, Jiangnan University, 1800 Lihu Road, Wuxi, Jiangsu 214122, China;

^2^ Science Center for Future Foods, Jiangnan University, 1800 Lihu Road, Wuxi, Jiangsu 214122, China;

^3^ Key Laboratory of Industrial Biotechnology, Ministry of Education and School of Biotechnology, Jiangnan University, 1800 Lihu Road, Wuxi, Jiangsu 214122, China;

^4^ Jiangsu Province Engineering Research Center of Food Synthetic Biotechnology, Jiangnan University, Wuxi 214122, China.

* Correspondence to:

Jingwen Zhou

Science Center for Future Foods, Jiangnan University, 1800 Lihu Rd, Wuxi, Jiangsu 214122, China.

Phone: +86-510-85914371, Fax: +86-510-85914371

E-mail: [zhmzhou@jiangnan.edu.cn](mailto:zhmzhou@jiangnan.edu.cn), zhoujw1982@jiangnan.edu.cn

**Supplementary Figures:**


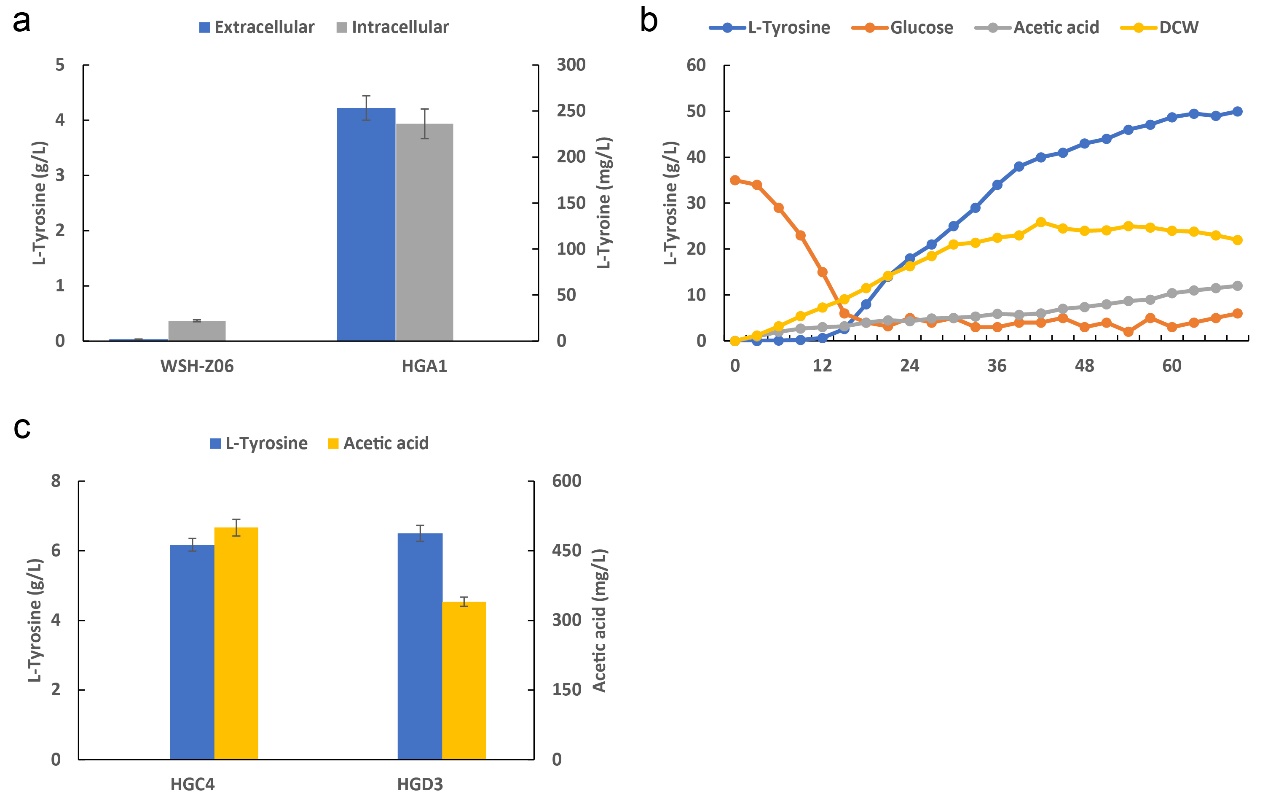


**Fig. S1. Supplementary charts for results 2 and 4.**

(a) Extracellular and extracellular L-tyrosine content of WSH-Z06 and HGA1. HGA1 can accumulate a large amount of L-tyrosine within 48 h of shaking, but the intracellular L-tyrosine level is 942% higher than that of the starting strain. (b) Fed-batch fermentation validation of HGC4 in a 5-L fermenter. After 69 h of fermentation, about 50.2 g/L of L-tyrosine was accumulated, but the growth of the strain was significantly inhibited. The highest OD_600_ was only about 61, which was significantly different from that of normal *E. coli*. (c) Effects of *poxB* knockout on the production of L-tyrosine and acetic acid. Knockout of *poxB* significantly decreased the acetic acid accumulation from 509 mg/L to 338 mg/L, which is a decrease of about 33.6%.


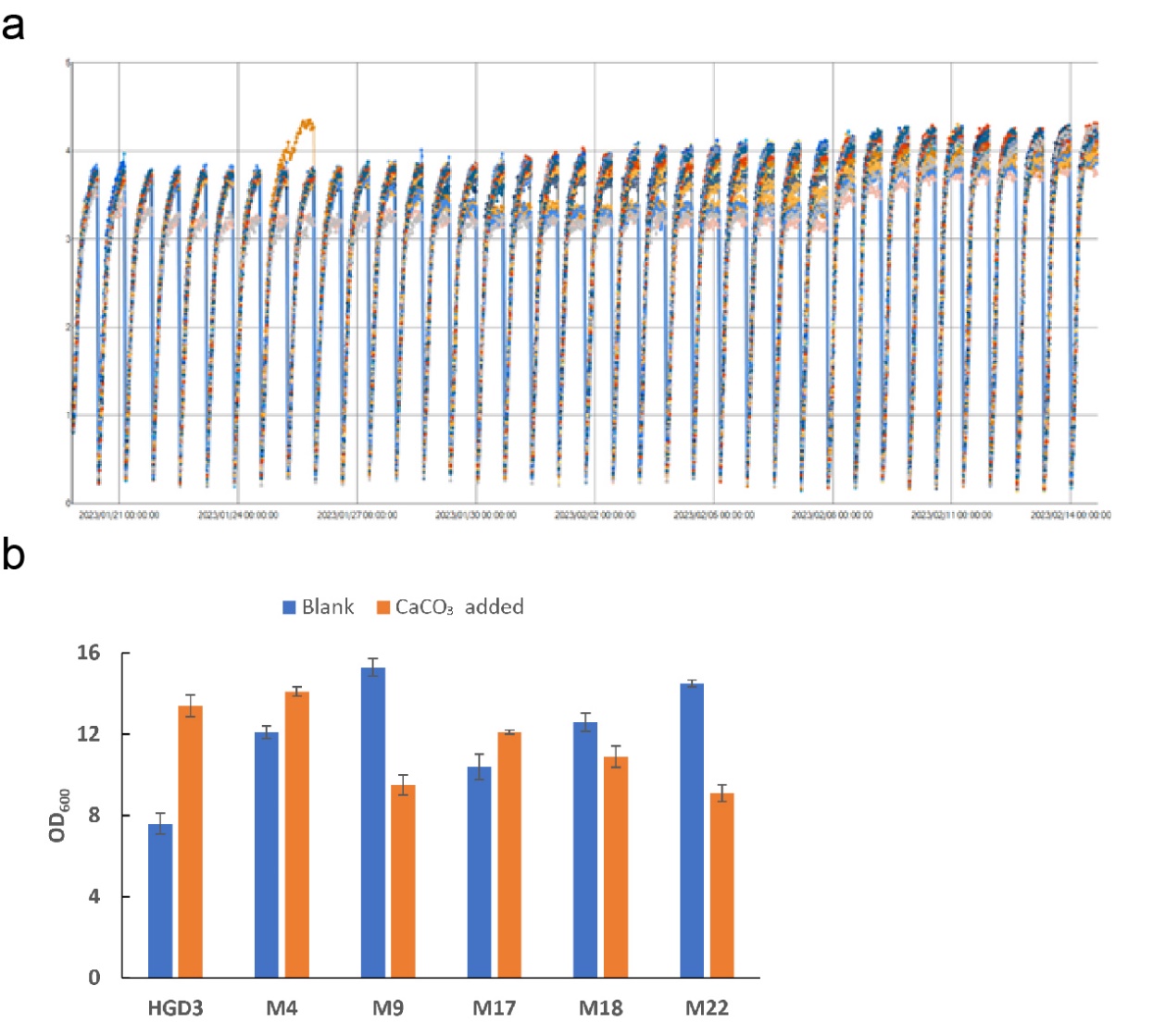


**Fig. S2. Generation and shake flask OD diagram of acid resistance evolution strains.**

(a) The evolution of *E. coli* in most droplets is relatively normal, and the strains in different droplets also show significant differences. However, in the last one third of evolution, some strains with poor adaptability previously screened showed significant growth recovery, and the reason has not been found yet. Therefore, at the end, these strains were excluded and only strains with normal evolutionary processes were selected for validation. (b) By rescreening the high-yielding strains screened from the plates in a shaking flask, it can be seen that the increase in OD has a significant effect on the accumulation of L-tyrosine.

**
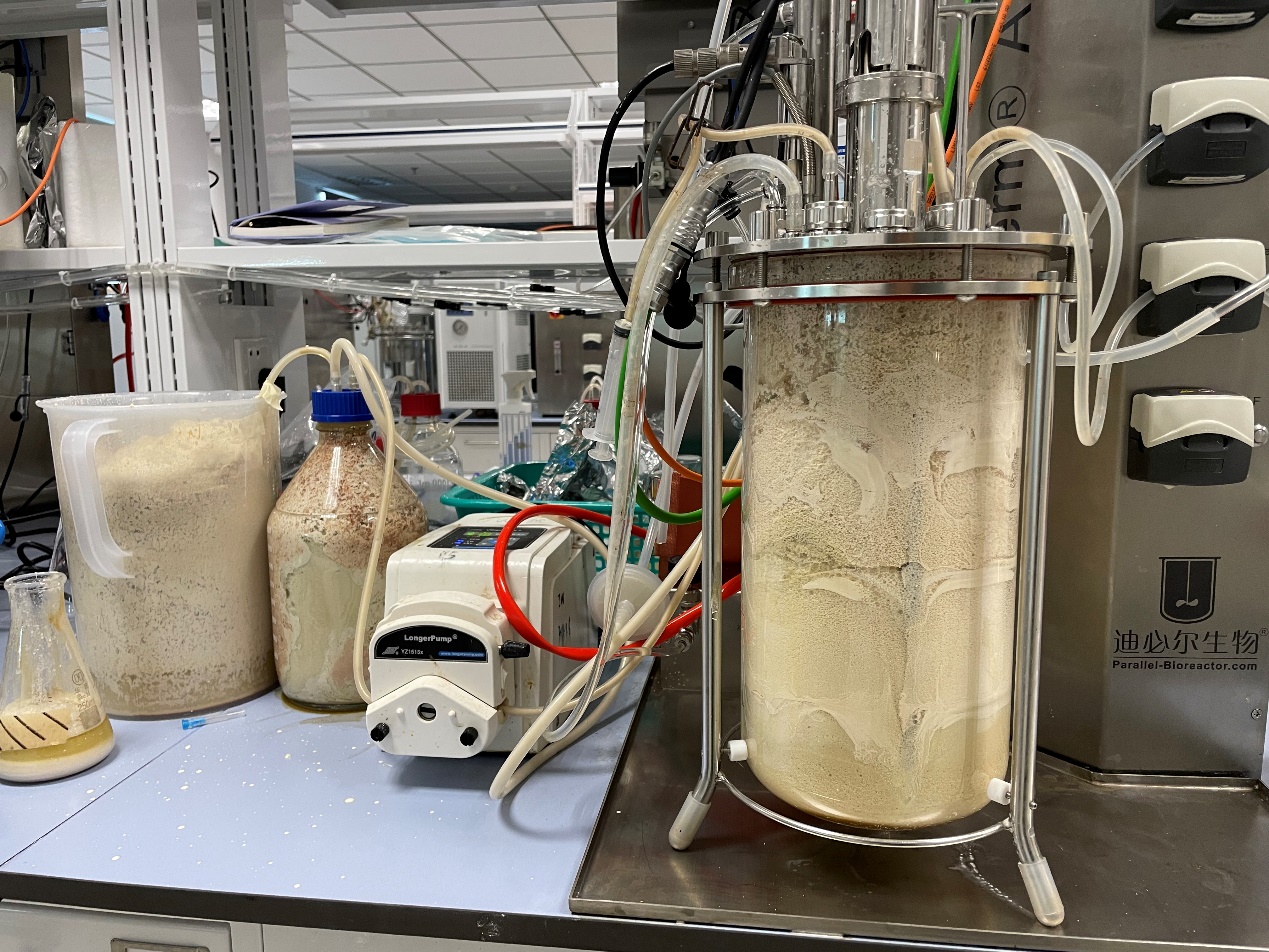
**

**Fig. S3. The reflux device.**

After 62 h of fermentation, a large amount of L-tyrosine in the fermentation tank was separated and attached to the inner wall of the fermentation tank, and a large amount of L-tyrosine was accumulated in foam collection bottles and waste collection bottles.

**Supplementary Tables:**

**Table S1 Nucleotide sequences of codon optimized genes in this study**

| **Gene** | **Sequence (5'-3')** |
| --- | --- |
| *aroG* | atgaattatcagaacgacgatttacgcatcaaagaaatcaaagagttacttcctcctgtcgcattgctggaaaaattccccgctactgaaaatgccgcgaatacggttgcccatgcccgaaaagcgatccataagatcctgaaaggtaatgatgatcgcctgttggttgtgattggcccatgctcaattcatgatcctgtcgcggcaaaagagtatgccactcgcttgctggcgctgcgtgaagagctgaaagatgagctggaaatcgtaatgcgcgtctattttgaaaagccgcgtaccacggtgggctggaaagggctgattaacgatccgcatatggataatagcttccagatcaacgacggtctgcgtatagcccgtaaattgctgcttgatattaacgacagcggtctgccagcggcaggtgagtttctcaatatgatcaccccacaatatctcgctgacctgatgagctggggcgcaattggcgcacgtaccaccgaatcgcaggtgcaccgcgaactggcatcagggctttcttgtccggtcggcttcaaaaatggcaccgacggtacgattaaagtggctatcgatgccattaatgccgccggtgcgccgcactgcttcctgtccgtaacgaaatgggggcattcggcgattgtgaataccagcggtaacggcgattgccatatcattctgcgcggcggtaaagagcctaactacagcgcgaagcacgttgctgaagtgaaagaagggctgaacaaagcaggcctgccagcacaggtgatgatcgatttcagccatgctaactcgtccaaacaattcaaaaagcagatggatgtttgtgctgacgtttgccagcagattgccggtggcgaaaaggccattattggcgtgatggtggaaagccatctggtggaaggcaatcagagcctcgagagcggggagccgctggcctacggtaagagcatcaccgatgcctgcatcggctgggaagataccgatgctctgttacgtcaactggcgaatgcagtaaaagcgcgtcgcgggtaa |
| *tyrA* | atggttgctgaattgaccgcattacgcgatcaaattgatgaagtcgataaagcgctgctgaatttattagcgaagcgtctggaactggttgctgaagtgggcgaggtgaaaagccgctttggactgcctatttatgttccggagcgcgaggcatctattttggcctcgcgtcgtgcagaggcggaagctctgggtgtaccgccagatctgattgaggatgttttgcgtcgggtgatgcgtgaatcttactccagtgaaaacgacaaaggatttaaaacactttgtccgtcactgcgtccggtggttatcgtcggcggtggcggtcagatgggacgcctgttcgagaagatgctgaccctctcgggttatcaggtgcggattctggagcaacatgactgggatcgagcggctgatattgttgccgatgccggaatggtgattgttagtgtgccaatccacgttactgagcaagttattggcaaattaccgcctttaccgaaagattgtattctggtcgatctggcatcagtgaaaaatgggccattacaggccatgctggtggcgcatgatggtccggtgctggggctacacccgatgttcggtccggacagcggtagcctggcaaagcaagttgtggtctggtgtgatggacgtaaaccggaagcataccaatggtttctggagcaaattcaggtctggggcgctcggctgcatcgtattagcgccgtcgagcacgatcagaatatggcgtttattcaggcactgcgccactttgctacttttgcttacgggctgcacctggcagaagaaaatgttcagcttgagcaacttctggcgctctcttcgccgatttaccgccttgagctggcgatggtcgggcgactgtttgctcaggatccgcagctttatgccgacatcattatgtcgtcagagcgtaatctggcgttaatcaaacgttactataagcgtttcggcgaggcgattgagttgctggagcagggcgataagcaggcgtttattgacagtttccgcaaggtggagcactggttcggcgattacgtacagcgttttcagagtgaaagccgcgtgttattgcgtcaggcgaatgacaatcgccagtaa |
| *fpk* | TTATTCATTGTCACCAGCAGTAGCAGCAGTAGCAGACAACATTTGTGTTTCATCAACCTTAACATCAGGGTATACCCAATCAGTAAACTCTGGAATGTCATAACCATTATCGACTGCGAATTGAAATGCTTTCTTTCTAAAAGCGTTTAATTCGTCGATCTTATCGGCGTATTTGTCTGCATCAATTAACTTCAAAGCGGCTGCTTGCAATGCATATCTGTCCATATCATTTACTCTGACCATATCAAAAGGTGTAGTGGTTGAACCTTGTTCTTTGTAACCGACAACGTGGAAATTGTCATGGTTTGGTCTATCATAGATCAAACCTCTAACATCTTGGGCGTAGGAATGATATGCAAACAATACTGGTTTATCAGCAGTAAACAATTCAGTAAACTCTTCGTCAGTCAATGCTTCATCGTTGTTTTCTCTTGATTGTAACTTCAACAAATCTACGACGTTAACTACTTTAAACTTAATACCCATTTTGTTCAAAGCATCACTAGCGGCCATCAATTCTTGTGTTGGGACGTCACCGGCGGATGCCAAGACAACTTGAACTTCATCGTTATTTTCTGCATTAGAAGCCCATTTCCATTCAGCAGCACCAGCTTCTAATTCAGCTCTGGCTTCATCCAAGGTAACCCATGTAGGTGCTGGTTGTTTACCGGCGAATATTGCATTGATTTTGTTAGTTGACTTGAAGCACTTTTCAGAGATAGCCAACAACATGTTAGCGTCAGTGGCAAAATAGATGTTGGTAACGTGATCGTTATTGAAGGTCTTATTAATCAACAATGATGTGACACCTGGGTCTTGGTGAGAAAAACCATTATGATCTTGTCTCCATACATGTGAAGAGACTAACAAGTTAACACTGGAGATAGGCTTTCTCCAAGGTATTTCTCTAACAGTAGCTTCTAACCATTTGGCGTGTTGGTTCAACATTGAATCGATAACATGTACAAAAGATTCGTATGAAGACCAAATACCATGTCTACCTGTTAACAAATATGCTTCCAAGAAACCTTCGCATTGGTGTTCTGATAATTGTTCTGTTACTTGACCAGTGACTGCCATGTGTTCATCAACTAAACCAGACAAGTAACCATTGTCCCATTGCTTATCGGTTACTTCATAAGTTGCGTTCAATCTGTTACTAGCTGTTTCATCAGGACCGAATATTCTAAAGGAGTCTGGGTTATTTTTAATGATATCTCTACAATATGCACCCAAAGCTCTAGGGGCTTCAACTTGACCCCAACCATGACCGTATTCCTTAACACCAGTTACTTCATATTGGTCTAATTCTGGCAATTTCAAATCTTCTCTGATAACACCACCATTAGCGTTAGGATTGGCACCTATTCTCAATTCACCCTTTGGCATAAAGGCTGTAACGTCATCTTTAATTGAACCATCTGCATTGAATAATTCTTCAGGCTTGTAAGATTCCATCCAACCTTTCAAGACTTCAAAGTGTTCTTCGGTATCTCTTGCACTAGCTAATGGAACTTGATGTGCTCTCCAGGAACCTTCTGTCTTTTTACCATCGATGAACTTAGGGCATGTCCAACCCTTTGGAGTTCTAAAAATCAACATAGGGTAGAATGGTCTAGTCATGTCATCGGTTTGAGCAGCAGCTTTTATGTCACAAATTTCATCGAAGATAGTTTCGAACAATTCAGCGAATCTTCTGTGGATAGACATGTGGTCTTCATTATCGAAACCGGCAACAAATTCGTAAGGGTGATAACCCATACCACGGAAAAAGTCATGTAATTCTTCATCAGATATTCTGGCCAAGATTGTTGGATTTGCAATCTTGTAACCGTTCAAATGCAAGATAGGCAATACGATACCATCAGTTCTTGGATTGACCAATTTGTTAGATTGCCAACCGGTAGCTAATGGACCTGTTTCGGCTTCACCGTCACCTATAATACAAGGAACAAACAATGATGGGTTATTCATTACTGCACCGTAAGCATGAGATAAAGCATAACCCAATTCACCACCTTCGTGAATTGAACCAGGGGTTTCTGGTGCAAAATGACTAGGGATACCACCTGGATAGGAGAATTGTCTGAAAAACTTTTGCAAACCAGCTTCATCTTTTGTTATGTTTGGGTAGTATTCAGTGTAAGTACCGTCAACATAACTTTGGGAAGTACCAGCTGGACCACCATGACCTGGACCCATTATAAAAACGGTGTTTTGTTGGTGATCAGCGATCAATCTGTTGATATGGGCCAACAAGAAGTTCAAACCTGGTGTAGTACCCCAGTGACCGACTAATCTATGCTTAACGTCATCTCTAGTAAAAGGTTCCTTCATCAATGGGTTACTTCTCAAGTAGATTTGACCTATGGACATATAGTTGGTAACTCTCCAATATTTATCCATACCTTCGATTGCTTCTTCTGAAACTGGTCTATCCAACTTTTGCCAAGGAGTACCGATTACAGGGTTAGTCAT |

**Table S2 Primers used in this study**

| **Primers** | **Sequence (5'-3')** |
| --- | --- |
| *tyrR*-SG-F | ccacgggacagtacgcacatgttttagagctagaaatagcaagttaaaataag |
| *tyrR*-SG-R | atgtgcgtactgtcccgtggactagtattatacctaggactgagctagctg |
| *tyrR*-U-F | cggaatcaacgttgatgattgcgg |
| *tyrR*-U-R | aggcatattcgcacttcggcgtaaagatatccg |
| *tyrR*-D-F | gccgaagtgcgaatatgcctgatggtgcaacacc |
| *tyrR*-D-R | gatctgtctgacgtcaccctcg |
| *pheA*-SG-F | ATATACCGAAAGTACGTCTGgttttagagctagaaatagcaagttaaaataaggctag |
| *pheA*-SG-R | CAGACGTACTTTCGGTATATactagtattatacctaggactgagctagctg |
| *pheA*-U-F | cgtctcgccaaactggaaaaatgg |
| *pheA*-U-R | cttttcaccccgatttgggaggccttattg |
| *pheA*-D-F | ctcccaaatcggggtgaaaaggtgccggatgatgtgaatcatcc |
| *pheA*-D-R | caatggtttctggagcaaattcaggtctg |
| *trpE*-SG-F | attgccggaacacgcccacggttttagagctagaaatagcaagttaaaataagg |
| *trpE*-SG-R | cgtgggcgtgttccggcaatactagtattatacctaggactgagctagctg |
| *trpE*-U-F | ccaggcgttcaattaaggtttgcg |
| *trpE*-U-R | gtttttatctcgccgaactgcgtcacgatcttgac |
| *trpE*-D-F | gacgcagttcggcgagataaaaacagaaatcagggcag |
| *trpE*-D-R | cgactctcgaactgctaacctgc |
| *adhE*-SG-F | AAGTATAAGAAGGAGATATACATATGAACATCTCTCGTCGTAAACTGCT |
| *adhE*-SG-R | AAGTATAAGAAGGAGATATACATATGCGTGATGGTAAATTCGTTGAATCTAAAAGC |
| *adhE*-U-F | GTGGCAGCAGCCTAGGTTAATTATTTTTTGAAACGATCCAGGCTGAACG |
| *adhE*-U-R | TTCGATTAAaaggagatataccATGCGTATCGATATCAAAGATTCCACC |
| *adhE*-D-F | TTCGATTAAaaggagatataccATGCGTATCGATATCAAAGATTCCACC |
| *adhE*-D-R | ttaagtataagaaggagatatacatatgAAAATCATCGCGTACGCGG |
| *pflB*-SG-F | CATggtatatctccttTTAATCGAATTTAACCTGGGTATCCGC |
| *pflB*-SG-R | CTACTGGCATTGTCGCTTCAgttttagagctagaaatagcaagtt |
| *ldhA*-SG-F | gatacgcgcggtgaatacgggttttagagctagaaatagcaag |
| *ldhA*-SG-R | ccgtattcaccgcgcgtatcactagtattatacctaggactgag |
| *ldhA*-U-F | cgctacatgcaggacaaacg |
| *ldhA*-U-R | gattgcgctgtgcccgaacgaactggttta |
| *ldhA*-D-F | gcggaaaccgaggactcgttcacctgttgc |
| *ldhA*-D-R | caaacgtccgtcaacaccac |
| *ackA*-SG-F | ggtcaccgtatcgtacacgggttttagagctagaaatagcaagttaaaataagg |
| *ackA*-SG-R | ccgtgtacgatacggtgaccactagtattatacctaggactgagctagctg |
| *ackA*-U-F | cagggcaatctgccagcagag |
| *ackA*-U-R | tcttcgttggcgtcagggagccatagagcgta |
| *ackA*-D-F | ctccctgacgccaacgaagaactggttatcgcg |
| *ackA*-D-R | gcgatttcgtagttcagagactgg |
| *pta*-SG-F | gtaaccgccagtcagcagcagttttagagctagaaatagcaagttaaaataagg |
| *pta*-SG-R | tgctgctgactggcggttacactagtattatacctaggactgagctagctg |
| *pta*-U-F | catgggtacccgttctggtgatatc |
| *pta*-U-R | tgatgacgaggctgagctggcggtgtgaaat |
| *pta*-D-F | ccagctcagcctcgtcatcatccgcagctttg |
| *pta*-D-R | ctgtcccgtatttcgatcctgagg |
| *poxB*-SG-F | ggtgaaaatagcgtcatcgggttttagagctagaaatagcaagttaaaataagg |
| *poxB*-SG-R | ccgatgacgctattttcaccactagtattatacctaggactgagctagctg |
| *poxB*-U-F | ggcaacactttgccgttgtgg |
| *poxB*-U-R | cataatcgccgaactggcgaaaacaaactggc |
| *poxB*-D-F | tcgccagttcggcgattatgcgagaaccaaatcc |
| *poxB*-D-R | cgatgagtggcgtaactatccgg |
| *tyrP*-SG-F | agaaaatatatgccaccagggttttagagctagaaatagcaagttaaaataagg |
| *tyrP*-SG-R | cctggtggcatatattttctactagtattatacctaggactgagctagctg |
| *tyrP*-U-F | cgacaaagaactctctaccctcgac |
| *tyrP*-U-R | GACTGAGCTAGCTGTCAAgctttcttctgtcctgacgatctttatg |
| *tyrP*-D-F | gcaaaagaactgctgtaagggtgatcagatagcctcaaattcc |
| *tyrP*-D-R | gaatctggcttttcaacatatggccg |
| *aroP*-SG-F | aatcaccacaaagaatacgggttttagagctagaaatagcaagttaaaataagg |
| *aroP*-SG-R | ccgtattctttgtggtgattactagtattatacctaggactgagctagctg |
| *aroP*-U-F | cgtgagtatttgcgtgagctgc |
| *aroP*-U-R | acgaggtttctctctctacgccctcacccg |
| *aroP*-D-F | cgtagagagagaaacctcgtgcggtggttg |
| *aroP*-D-R | ggtcttaccaatttcatgtctgtgacg |
| *dadX*-*cvrA*-SG-F | ggcgaagaatatcatccatggttttagagctagaaatagcaagttaaaataagg |
| *dadX-cvrA*-SG-R | catggatgatattcttcgccactagtattatacctaggactgagctagctg |
| *dadX-cvrA-*U-F | gtgatatcgccaataccggattacg |
| *dadX-cvrA*-U-R | GGACTGAGCTAGCTGTCAAtgcggtgagttcaggttccgg |
| *dadX-cvrA*-D-F | gcaaaagaactgctgtaacaggcgttctacataaaacgcttacgc |
| *dadX-cvrA*-D-R | ggcgatgtgttgtgtgtaattgg |
| *yeeJ-yeeL*-SG-F | ctttgccccaggaaaaacgggttttagagctagaaatagcaagttaaaataagg |
| *yeeJ-yeeL*-SG-R | ccgtttttcctggggcaaagactagtattatacctaggactgagctagctg |
| *yeeJ-yeeL*-U-F | ctcttcaggtcaggctccagtc |
| *yeeJ-yeeL*-U-R | cgtcgtggttaactttagatcaaagcagtactcaccg |
| *yeeJ-yeeL*-D-F | gatttcgcggctggttttctgtgatgattgaccag |
| *yeeJ-yeeL*-D-R | cctgttattcgggcgcttgaattg |
| *ykgH-betA*-SG-F | ggtgaaaacgactatcacgggttttagagctagaaatagcaagttaaaataagg |
| *ykgH-betA*-SG-R | ccgtgatagtcgttttcaccactagtattatacctaggactgagctagctg |
| *ykgH-betA*-U-F | gtatctcatcgagaacttgcctgcc |
| *ykgH-betA*-U-R | GACTGAGCTAGCTGTCAAaccgttccagagagggggacc |
| *ykgH-betA*-D-F | gaagaaataaaactctcaatctgatcggttcctgc |
| *ykgH-betA*-D-R | gtgcggattaaatcccgcgac |
| *tktA-*F | TTGACAGCTAGCTCAGTCCTAGGTATAATACTAGTAAAGAGGAGAAAAAGCTTgcgagtagattgcgcaacatgc |
| *tktA*-R | ttacagcagttcttttgctttcg |
| *ppsA*-F | TTGACAGCTAGCTCAGTCCTAGGTATAATACTAGTAAAGAGGAGAAAAAGCTTcatccggttaaatatgcaaagataaatgcg |
| *ppsA*-R | ttatttcttcagttcagccaggcttaacc |
| *yddG*-F | TTGACAGCTAGCTCAGTCCTAGGTATAATACTAGTAAAGAGGAGAAAAAGCTT atgacacgacaaaaagcaacgc |
| *yddG*-R | ttaaccacgacgtgtcgccag |
| *pntAB*-F | TTGACAGCTAGCTCAGTCCTAGGTATAATACTAGTAAAGAGGAGAAAAAGCTT atgcgaattggcataccaagagaac |
| *pntAB-*R | ttacagagctttcaggattgcatcc |
| *udhA*-F | TTGACAGCTAGCTCAGTCCTAGGTATAATACTAGTAAAGAGGAGAAAAAGCTTatgccacattcctacgattacgatg |
| *udhA*-R | ttaaaacaggcggtttaaaccgtttaacg |
